# Active Shooter Training Drill Increases Blood and Salivary Markers of Stress

**DOI:** 10.3390/ijerph17145042

**Published:** 2020-07-13

**Authors:** Matthew J. McAllister, M. Hunter Martaindale, Liliana I. Rentería

**Affiliations:** 1 Metabolic & Applied Physiology Laboratory, Department of Health & Human Performance, Texas State University, San Marcos, TX 78666, USA; lillie@txstate.edu; 2 ALERRT Center, Texas State University, San Marcos, TX 78666, USA; Hunter.Martaindale@txstate.edu

**Keywords:** police, adrenaline, hormones, law enforcement, tactical, cortisol, scenario training, sympathoadrenal axes, cardiometabolic health

## Abstract

Police officers are frequently engaged in a variety of high-stress scenarios, such as high-speed chases and other suspect conflicts, that cause significant increases in a variety of physiological and psychological stress markers. The purpose of this study was to investigate salivary and blood markers of stress in response to an active shooter training drill (ASD). Thirty-one participants (n = 31; males = 15, females = 16; Age: 21 ± 3.5 years) participated in an ASD involving professional actors playing the role of one active gunman, as well as four victims. The ASD lasted approximately 50 s. Blood samples were collected 15 min prior as well as after the ASD and analyzed for epinephrine (EPI) and norepinephrine (NE) levels. Saliva samples were collected 30 and 5 min prior to the ASD and 5 and 30 min after the ASD, were analyzed for cortisol, α-amylase, and secretory immunoglobulin-A (SigA). The ASD resulted in significant (*p* < 0.05) increases in EPI, α-amylase, and SigA levels. The increase in NE from pre to post ASD approached significance (*p* = 0.06). These results demonstrate that a short duration (~50 s) ASD results in significant increases in both blood and salivary markers of stress. These data may provide meaningful implications for those engaged in high-stress tactical occupations, especially law enforcement and military personnel, as chronic exposure to such occupational stressors can contribute to cardiometabolic disease.

## 1. Introduction

Law enforcement officers are exposed to a variety of acute and chronic psychological stressors that result in the activation of the hypothalamic pituitary adrenal (HPA) [1,2] and sympathoadrenal (SA) [3] axes. The result of the activation of HPA and SA axes is elevated secretion of stress hormones and neurotransmitters such as cortisol, epinephrine, and norepinephrine [4]. Chronic strain on the HPA and SA axes can result in chronic elevations of stress hormones and may lead to increased risk for developing a number of cardiometabolic diseases [4]. To illustrate this, metabolic syndrome is largely prevalent in law enforcement officers and is likely associated with physical inactivity [5]. In addition, even clinically healthy police officers have been shown to have elevated rates of atherosclerosis which was not dependent upon traditional cardiovascular disease risk factors [6].

Police officers are frequently engaged in a variety of high-stress scenarios, such as high-speed chases and other suspect conflicts, that directly impact their life and/or well-being [7,8]. Other occupational stressors such as shift work, high performance expectations, and unpredictability of potential exposure to violence contribute to police work being identified as one of the most stressful occupations [9,10]. These stressors can also impact psychological health and may contribute to increased rates of posttraumatic stress disorder [11], depression [12], and suicide attempts [13].

Several studies have demonstrated elevated levels of stress markers such as blood and salivary cortisol, as well as subjective anxiety in response to acute occupational stressors in a variety of simulated policing activities with and without the threat of a firearm [8,9,14,15,16]. Video-based workplace scenarios have also been shown to cause significant increases in salivary stress markers including cortisol, interleukin-6, α-amylase, and secretory immunoglobulin A in police officers [9]. While salivary and blood levels of cortisol reflect activation of the HPA axis, salivary α-amylase reflects activation of the SA axis [17]. It has been suggested that elevated cortisol levels are associated with higher levels of physical performance in law enforcement [14]. However, worth noting is that chronic activation of the HPA axis results in hypercortisolemia itself, which can lead to obesity and cardiometabolic diseases [4]. These findings suggest that elevated cortisol levels may be associated with improved performance while exhibiting negative health outcomes. It is possible that cortisol is not responsible for the improvement in performance per se, but rather an effect of increased physiological arousal. However, further research is needed to parse out the positive and negative outcomes of elevated cortisol levels for law enforcement.

While police work has shown to increase a number of physiological and psychological stress markers [8,9,14,15,16,18], to our knowledge, only one study has investigated the impact of active shooter training on markers of psychobiological stress [15]. Potentially important to note is that the active shooter training involved officers which were given different roles, and the total scenario lasted approximately 15–20 min [15]. It was reported that officer position and/or involvement during training scenarios impacted psychobiological stress measurements [15], suggesting that the role of each officer has an impact, and perhaps direct involvement or engagement with a suspect or a life-threatening scenario is most stressful in training. Interestingly, a similar study also reported that potentially life-threatening training scenarios with a gunman induces greater elevations in salivary stress markers than non-life-threatening training scenarios [9]. It is important to note that this study used video-based scenarios projected on a wall for the participants, and showed that a motorcycle chase training scenario lasting only two minutes causes significant increases in salivary stress markers [9]. Therefore, the magnitude of stress response to these scenarios does not appear to be dependent upon the duration, but the engagement and potential for a life-threatening scenario.

While prior research has examined the physiological response to shoot/no-shoot scenarios by a single student recruit in non-realistic scenarios [14] or a pair of officers responding to a robbery as a team [18], and one has examined long-duration active shooter scenarios [15], none have sought to understand realistic short-duration active shooter scenarios. We aimed to fill this gap in the literature. As such, the purpose of this study was to determine the impact of a realistic, short-duration active shooter training scenario on the salivary and blood markers of stress. We hypothesized that both blood and salivary stress markers would significantly increase in response to the active shooter training.

## 2. Materials and Methods

The experimental procedures performed were in line with Helsinki and reviewed and approved by the University’s Institutional Review Board. Participants (n = 31; 15 males, 16 females) were current university students that were recruited from the university campus via word of mouth to participate in this study. Each participant was required to provide electronic informed consent prior to filling out a health history questionnaire, as well as a lifestyle/wellness questionnaire. Participants were required to be: (1) non-cigarette smokers, (2) free from donating blood in the last 30 days, and (3) free from any major psychological stressor in the last 30 days such as death in the family, job change. Participants also indicated if they were physically active by participating in exercise at least three times per week. Lastly, participants were asked to arrive having fasted for at least 4 h for the experimental testing session.

### 2.1. Active Shooter Training Drill

The active shooter training drill (ASD) lasted ~50 s and involved a total of five professional actors filling the roles of one active shooter with a firearm, as well as four victims. Actors were used to make the scenario as close to reality as possible. Victims wore a variety of moulage (realistic fake wounds) and realistic fake blood to increase authenticity.

Participants were asked to sit at rest in a quiet bunker room for 30 min before being transported to the building in which the ASD took place. After the resting period, participants were transported a short distance (~90 m) to the ASD facility. During the transportation period, participants were told they were playing the role of the first responding police officer to a “shots fired” call. Participants were informed that only a limited amount of information would be given to them in the form of dispatch radio traffic to simulate the amount of information a real police officer receives. The researcher further informed each participant that he would tell him/her when they could enter the scenario to simulate arrival time. Participants were allowed to acclimate to the Glock 17T training pistol prior to the ASD to ensure they were comfortable in its operation. Once the participant indicated he/she was ready, the researcher played the simulated radio traffic to start the ASD. Approximately 20 s into the simulated radio traffic, the active shooter began firing his blank gun inside the building. Approximately 5 s later, a simulated fire alarm activated, additional shots were fired, and the victims began to scream. At this point, the researcher would inform the participant that they have arrived on the location, and they could enter the building.

Immediately upon entering the hallway, participants were confronted with one victim laying on the floor. The victim exhibited traumatic injuries (i.e., eviscerated bowels, gunshot wound to her upper thigh, a pool of blood on the floor). After the participant passed the first screaming victim, a second victim ran out of the scenario room approximately 7 feet down the hall towards the participant. This victim exhibited traumatic, but nonlife-threatening injuries (i.e., gunshot wound to her upper arm and leg). As this victim ran past, she screamed for help. The participant would then advance the last few feet to the ASD room. From the ASD room threshold, the participant would observe one victim on the ground with a traumatic head injury, laying in a pool of blood. Furthermore, the participant would witness the last victim collapse after the shooter fired multiple shots. At this point, the shooter would either fall to the ground if the participant fired his/her training weapon or turn to the participant to elicit the participant to fire his/her training weapon. The researcher promptly ended the scenario, retrieved the training weapon from the participant, and escorted the participant back to the bunker. The researcher explained the purpose of the study and asked each participant what they experienced. No participant reported the ASD as traumatic; however, if any participants voiced (or appeared to have experienced) potential trauma, the researcher would have directed them to the mental health services provided through the university. These mental health services were also listed on the IRB approved informed consent form. Testing took place between the hours of 09:00 and 15:00.

### 2.2. Saliva and Blood Sample Collection and Analysis

Saliva samples were collected both 30 and five minutes prior to the ASD, as well as five and 30 min after ASD. Saliva samples were collected via passive drool technique using a collection tube (Salimetrics, PA, USA). Participants were initially asked to rinse their mouth with water for approximately 30 s, 10 min prior to providing a saliva sample. During the collection period, participants were asked to tilt their head forward and allow saliva to collect into a polypropylene vial until at least 500 μL was collected (approximately 2 min was required). Saliva samples were immediately stored on dry ice and subsequently stored at −80 °C until analysis.

Blood samples were collected 15 min before as well as 15 min after the ASD from an antecubital vein using a 21g butterfly needle with a push button safety retraction. A total of 20 mL was collected into sealed vacutainers containing sodium heparin and immediately cooled on ice and centrifuged at 1600x *g*. Plasma was aliquoted and stored at −80 °C until subsequent analysis.

Saliva samples were thawed and analyzed in duplicate for cortisol (S-CORT), α-amylase, secretory immunoglobulin-A (SigA) using commercially available kits (Salimetrics, PA, USA). In terms of sample volume, 8 μL of diluted saliva (1:10) was used for α-amylase analysis, 10 μL of diluted saliva (1:5) was used for SIgA analysis, and 25 mL was used for S-CORT analysis. Plasma samples were thawed and analyzed for epinephrine (EPI) and norepinephrine (NE) (300 μL) using an enzyme-linked immunosorbent assay (ELISA) which included an extraction procedure (ALPCO, NH, USA). Each sample was analyzed in duplicate. Absorbance was read using EPOCH II plate reader (Biotek, Winooski, VT, USA). Note, α-amylase is a kinetic assay which requires 37 °C incubation, for which a standard laboratory incubator was used (Thermo Fisher Scientific, Waltham, MA, USA).

### 2.3. Statistical Analysis

Statistical procedures were conducted with SAS v 9.4 (Cary, NC, USA). Changes across time for salivary measures were determined with one-way repeated measures ANOVAs. In the instance of a significant main effect, Tukey post hoc tests were conducted to compare means. In the instance of a significant main effect (*p* < 0.05), effect sizes were calculated as partial eta squared (η^2^). Blood levels of EPI and NE pre and post ASD were analyzed with paired *t*-tests. Effect sizes for pre- and post-ASD levels of EPI and NE were analyzed using Cohen’s *d*.

## 3. Results

Data are reported as mean ± standard deviation unless otherwise specified. Participant-descriptive characteristics are shown in Table 1. Saliva samples were collected from all 31 participants (n = 31), however, blood samples were collected from 25 participants (n = 25), as some participants requested to avoid a blood draw due to needle phobia.

### 3.1. Salivary Measures

**Figure 1:**
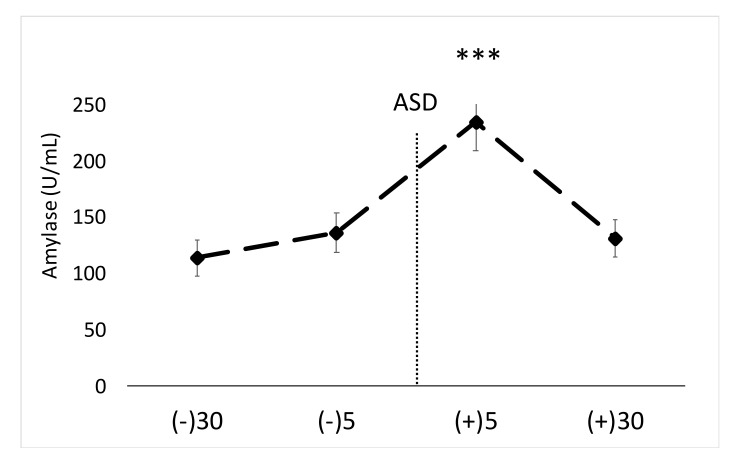
Data are shown as mean ± SE. Changes in salivary α-amylase across time. ASD = active shooter training drill. (−30) = 30 min prior to ASD, (−5) = 5 min prior to ASD, (+5) = five min post-ASD, (+30) = 30 min post-ASD. *** Denotes significant (*p* < 0.001) increase compared to all other time points.

With respect to salivary α-amylase, a significant main effect was noted (F = 20.94, *p* < 0.01, η^2^ = 0.41). No change was noted between −30 min and −5 min pre ASD, however the five min post-timepoint demonstrated significantly elevated α-amylase activity compared to all other timepoints (*p* < 0.001). α-Amylase data can be seen in Figure 1. The average intra-assay %CV for this assay was ~4.5%. The inter-assay %CV for this assay was 5.0%.

**Figure 2:**
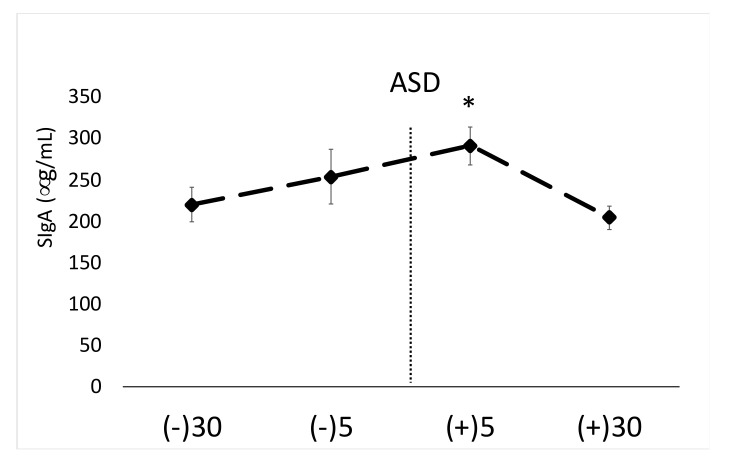
Data are shown as mean ± SE. Changes in salivary secreted immunoglobulin A (SIgA) across time. ASD = active shooter training drill. (−30) = 30 min prior to ASD, (−5) = 5 min prior to ASD, (+5) = five min post-ASD, (+30) = 30 min post-ASD. * Denotes significant (*p* < 0.05) increase compared to 30 min post-stress.

A significant main effect for time was noted for SigA (F = 3.62, *p* = 0.01, η^2^ = 0.10). Post hoc results demonstrated no change between −30 and −5 min pre ASD; however, SigA levels were significantly elevated +5 min post ASD in comparison to 30 min post-stress. The increase from 30 min pre-ASD to five min post-ASD approached significance (*p* = 0.07). Mean SigA data can be found in Figure 2. The average intra-assay %CV for this assay was <5.9%. The inter-assay %CV for this assay was 6.1%.

**Figure 3:**
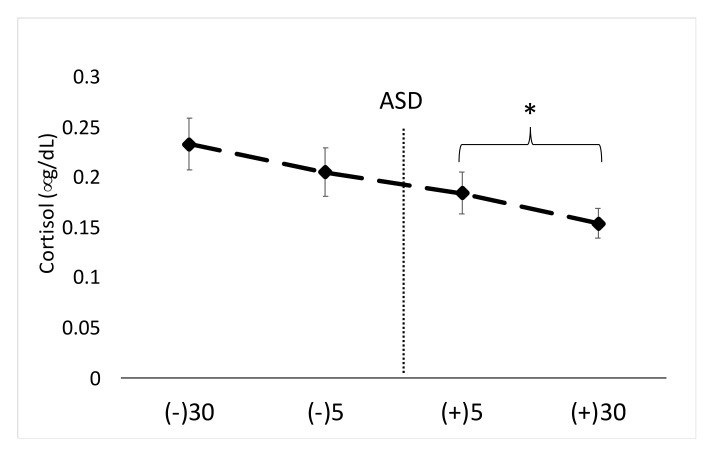
Data are shown as mean ± SE. Changes in salivary cortisol across time. ASD = active shooter training drill. (−30) = 30 min prior to ASD, (−5) = 5 min prior to ASD, (+5) = five min post-ASD, (+30) = 30 min post-ASD. * Denotes significantly (*p* < 0.05) lower values compared to −30 min pre-ASD.

With regard to mean S-CORT levels, a significant main effect for time was noted (F = 7.52, *p* < 0.001, η^2^ = 0.20). Post hoc results demonstrate a significant decrease in S-CORT levels at +5 min and +30 min compared to −30 min pre ASD. Changes in S-CORT levels can be seen in Figure 3. The intra-assay %CV for this assay was 7.2%. The inter-assay %CV for this assay as 6.1%.

### 3.2. Blood EPI and NE

**Figure 4:**
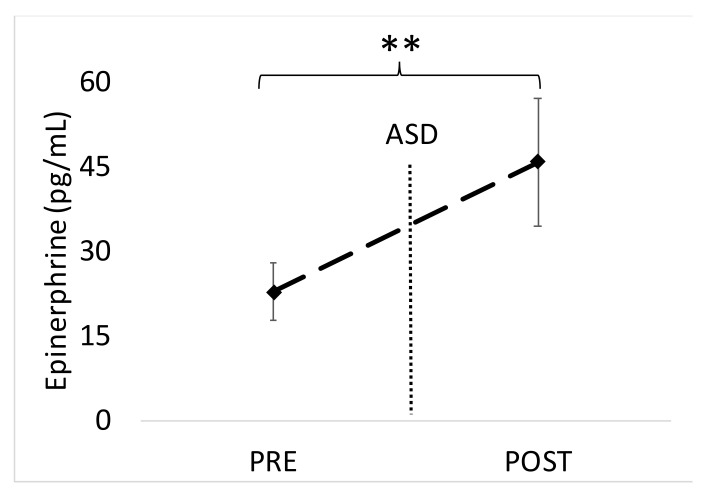
Data are shown as mean ± SD. Changes in blood epinephrine levels before and after the active shooter training drill (ASD). Pre = 15 min before the ASD, Post = 15 min after the ASD. ** Denotes a significant (*p* < 0.01) increase from 15 min pre- to 15 min post-ASD.

**Figure 5:**
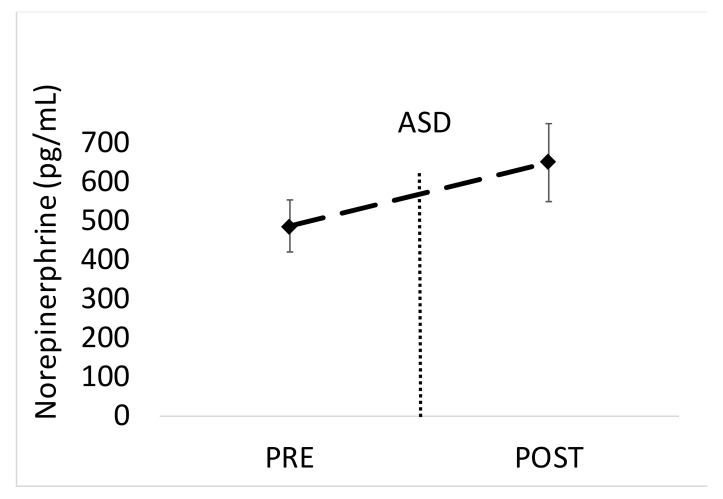
Data are shown as mean ± SD. Changes in blood norepinephrine levels before and after the active shooter training drill (ASD). Pre = 15 min before the ASD, Post = 15 min after the ASD.

There was a significant increase in blood EPI from 15 min pre- to 15 min post-ASD (*p* = 0.009, Cohen’s *d* = 0.68). Mean EPI data are shown in Figure 4. The increase in NE from 15 min pre- to 15 min post-ASD approached significance (*p* = 0.064, Cohen’s *d* = 0.47). Mean NE data are shown in Figure 5. The average %CV for these assays were 20%.

## 4. Discussion

The main findings of this study are that the active shooter training results in significant increases in biomarkers of physiological stress, namely salivary α-amylase, SIgA and blood EPI. While other studies have shown acute stress to facilitate increases in similar markers [8,9,14,15,16], the present findings are unique in that a real-life scenario was carried out by professional actors. Secondly, the ASD lasted less than one minute in duration (~50 s). Given the magnitude of biochemical stress that was induced by this short training drill, these results provide additional insight as to the potential stress that is imposed upon those working in high-stress tactical occupations, especially individuals working in law enforcement and military. Chronic exposure to such occupational scenarios can increase risk for developing cardiometabolic disease in police officers [4,12]. Therefore, the implications may not only be meaningful for law enforcement but potentially military personnel as well. However, it should be noted that law enforcement officers may differ in their physiological response based on their experience level with training and/or responding to ASD scenarios.

Previous research has shown police work causes significant increases in a variety of stress markers [8,9,14,15,16]. Most of these studies have shown increases in salivary stress markers in response to training scenarios such as force-on-force handgun training [16], simulated school shooting [15], and video-based simulated critical incident scenarios [9]. The results of the current study are similar to the aforementioned, as we reported significant increases in salivary stress markers α-amylase and SigA; however, to our knowledge we were the first to report blood and salivary stress markers in response to ASD. One recent study [15] investigated the impact of simulated school shooter training on psychobiological stress markers in police officers and reported increases in salivary α-amylase activity and psychological stress. Our results are similar in two ways: (1) we also found significant increases in salivary α-amylase activity, and (2) the present findings also showed the highest S-CORT levels before the training scenario started. In regards to S-CORT, both of these findings are perhaps attributed to the fact that cortisol is a slow-acting hormone that can be impacted by time of day and a number of confounding factors [19]. In order to detect changes in cortisol levels in response to acute psychological and/or physical stress, it is generally ideal to have the participants rest quietly and attempt to avoid exposure to stressors for at least 60 min prior to an intervention [20]. It is also possible that participants may anticipate psychological stress in a laboratory or field setting and fail to establish a baseline for cortisol measurement due to anticipation [15,21]. Considering these factors, attention should also be drawn to the finding that, while the acute ASD resulted in significant increases in physiological stress markers, the “recovery” appears to be relatively rapid, as salivary SigA and α-amylase were back to baseline levels within 30 min post-ASD. This could potentially be attributed to the fact that this was a short-duration psychological stress. Perhaps if the duration would have been longer, and/or if both psychological and physiological stress was imposed concurrently, the recovery would not be as rapid [4,19].

The findings from Strahler and Ziegert [15] also demonstrate that the psychobiological stress response in police officers depends on the position and direct roles of the officers involved. Specifically, officers that secured the front of a building during a simulated school shooter scenario demonstrated the highest levels of salivary stress markers including higher salivary α-amylase activity compared to officers that secured the sides and back of the building [15]. Similarly, Groer et al., [9] studied salivary stress and inflammatory responses in police officers engaged in two video-based training scenarios. Scenario one involved a motorcycle chase and stop lasting approximately two minutes, while scenario two involved a tactical active shooter search and clear drill lasting approximately six minutes. While both scenarios resulted in significant increases in stress markers, the scenario with an active shooter resulted in larger changes in salivary stress markers compared to those resulting from the motorcycle chase—such as S-CORT, and α-amylase [9]. Significant increases in α-amylase and SigA were noted in the current study, despite the short duration of the drill (~50 s), which again provides additional support that the nature of the training scenario is an important factor determining the stress response (i.e., life threatening, active shooter based).

Salivary α-amylase is suggested to be an indicator of psychological stress as a reflection of sympathetic activity or activation of the SA axis [22], while S-CORT activity is an indicator of the activation of the HPA axis [23]. These findings have led to increased trials incorporating salivary stress markers as opposed to blood markers, due to the non-invasive nature of collection. While several studies have reported a relationship between S-CORT, blood cortisol, NE, and salivary α-amylase activity in response to acute stress, there may not be a strong correlation between NE and α-amylase, despite the fact that both of these measures increase in response to acute stress [22].

The current study was limited by a number of factors which should be considered. First, the time of day was not uniform for each participant which may have impacted some of the results, especially S-CORT. Furthermore, S-CORT levels may have been impacted by the awakening response and this should be viewed as a limitation. However, the finding that S-CORT was highest prior to the ASD is not surprising, especially considering the similarity to other work [15]. It should be noted, however, that the variation between participants in terms of time of day for testing may have impacted S-CORT results. Moreover, both males and females were involved in this study, which is also commonly done [9]; however, since menstrual cycle phase can impact cortisol responses to a stressor [24], this could also be viewed as a limitation. Finally, it is important to note that the participants involved were not professional law enforcement officers, which may have an impact on the outcome variables. Participants were demographically similar to new law enforcement recruits (e.g., mean age = 22, physically active); however, participants in this study are unable to give insight into the performance of chronically stressed veteran law enforcement officers.

## 5. Conclusions

To the best of our knowledge, this was the first study to report increases in both blood and salivary markers of stress in response to ASD. These findings are meaningful given the brief nature of the ASD protocol and provide implications for tactical high-stress occupations that may train or be exposed to potentially life-threatening scenarios. Chronic exposure to scenarios causing increases in blood and salivary stress markers can cause increased risk of developing cardiometabolic disease [4,12]. Future work should incorporate similar protocols involving professional law enforcement officers and investigate the effect of various training interventions on the impact of acute ASD.

## Figures and Tables

**Table 1:** Participant Demographics.

Demographic	Data
Age ^1^	21.9 ± 3.5
Male	n = 15
Female	n = 16
Physically Active 3x/week	24 yes; 7 no

^1.^ Age is reported as mean ± SD.
